# Topical Insights Into the Post-Approval Controversies of Aducanumab

**DOI:** 10.3389/fphar.2021.787303

**Published:** 2021-11-18

**Authors:** Dhiraj Kumar, Dharmendra Kumar Yadav, Md Imtaiyaz Hassan

**Affiliations:** ^1^ Centre for Interdisciplinary Research in Basic Sciences, Jamia Millia Islamia, New Delhi, India; ^2^ College of Pharmacy, Gachon University of Medicine and Science, Incheon, South Korea

**Keywords:** Alzheimer’s drug, Aducanumab, Neurodegenerative diseases, FDA—Food and drug administration, Neuropsychological

## Background

The testimony of advocacy groups and patients offered strong grounds for regulatory flexibility in dealing with the unmet need for a disease-modifying therapy for Alzheimer’s disease (AD) (Fillit and Green, 2021). The U.S. Food and Drug Administration (FDA) chose to employ its “accelerated approval” method instead of giving the medication a conventional approval, usually reserved for drugs that benefit in big phase III trials. This is for therapies that are “reasonably likely” to assist patients but not certain. The Biogen first sought clearance for this medication based on a retrospective study of a subgroup of patients who took part in phase 3 trials, a potentially biased method. Such retrospective studies are appropriate for developing testable hypotheses for prospective trials, but they do not provide unbiased, compelling evidence to base effectiveness findings. The decision disregarded key aspects of the scientific process and risks eroding public trust in research, regulatory science and the FDA (Karlawish and Grill, 2021). However, according to Eric Siemers, a drug-development consultant in Zionsville, Indiana, Aducanumab’s clearance might spur additional investment and innovation into clinical research.

### FDA’s Decision Spaced Between Hope and Threat

Reduced amyloid plaques are an unvalidated and controversial measure of a drug’s action, which is one reason for the decision’s backlash. The amyloid reduction has not resulted in cognitive improvements in big trials of Alzheimer’s medication possibilities, which has made it a stumbling block for researchers. Aducanumab’s FDA approval procedure, as well as Aduhelm’s label, was both faulty. The too-broad wording merely says, “ADUHELM is indicated for the treatment of AD,” at the time of writing. The label does not specify whether patients must have Aβ pathology to be eligible for therapy, nor does it give any other information about the individuals who should get the medication. Clinicians and insurers are now faced with determining whether patients’ treatments are acceptable and essential. A lack of rigor in the approval of aducanumab for AD might result in catastrophic consequences (Perlmutter, 2021).

However, a high-ranking FDA official, Patrizia Cavazzoni, has acknowledged aducanumab’s larger impact. In a news conference, she added, “The accelerated approval process has been a very valuable instrument.” “We believe it serves as a model that may be used for other neurodegenerative diseases,” says the team. A medication that reduces dementia’s increasing impairments would be transformative for the millions of people living with AD and their care. Since the introduction of more than 20 authorized disease-modifying therapies for multiple sclerosis followed the expedited approval of interferon-β in 1993, which was based partly on data acquired by magnetic resonance imaging (MRI) that were reasonably expected to be predictive of clinical efficacy. In the aftermath of aducanumab, there’s no reason to expect anything less (Cummings, 2021). As a result, proponents of the FDA’s decision repeatedly express two emotions: despair and optimism. One essential feeling, however, has been conspicuously lacking from this discussion: trust. The full impact of the FDA’s decision on aducanumab is uncertain. Still, any future congressional hearings on NIH funding, the FDA’s decision, and Medicare reimbursement for Aduhelm would all show a negative influence on faith in the agency. Desperate need for the patients despite complex data set, post-approval rippling effect on the market and future drug development strategy, and the drug’s post-marketing trials to validate cognition efficacy are crucial landmarks of aducanumab’s approval. Reinstating that desperation should be the driving force for research funding, not how we perceive science and confound the research community (Mullard, 2021b).

## Raised Challenges for the Clinicians and Health Care Systems

Clinicians will face additional challenges as a result of Aducanumab. The use of biomarkers to provide an accurate diagnosis is crucial. Dose titration, surveillance for amyloid-related imaging abnormalities, and conveying realistic treatment goals will all be necessary. Aducanumab will put further strain on healthcare systems. Early identification of memory loss has not previously been a priority in medical care systems, but it now must be. New treatments are only worthwhile if they reach patients who can benefit from them. Once identified, treatment candidates will need a diagnostic evaluation and confirmation of amyloid presence by amyloid imaging or lumbar puncture with cerebrospinal fluid analysis. MRI monitoring will be required, and genotyping may be needed to assist in forecasting the likelihood of amyloid-related imaging abnormalities. Despite continued controversy about the FDA’s approval of Biogen’s aducanumab for AD, drug developers embrace the newly paved path to market for amyloid-lowering drugs.

## Urgent Need of Balancing Solutions

For patients, Aducanumab changed everything. It’s a hope for individuals with early AD that their illness may improve or be halted, and the inevitable descent towards insanity can be slowed. Dementia’s gradual unknowing will be postponed. Therefore, its approval is being hailed as a victory for patients. However, the U.S. healthcare system is unprepared for Aduhelm’s dubious efficacy, unrestricted market access, and high price. Being a novel treatment, it will drive up healthcare prices. Federal health programmers, commercial insurers, pharmaceutical firms, and other stakeholders will need to work together to create solutions that encourage innovation, ensure treatment equality for all patients who may benefit, and fit within inelastic budgets. Aduhelm can also cause brain swelling and bleeding, and patients may be exposed for years without the benefit of a costly serial MRI to check for them. Market access, regulation, and payment are all linked. A solution that balances expanded market access, patient safeguards, outcomes, and costs must be developed (Editorial, 2021).

## Impact on Other Neurodegenerative Diseases

**FIGURE 1:**
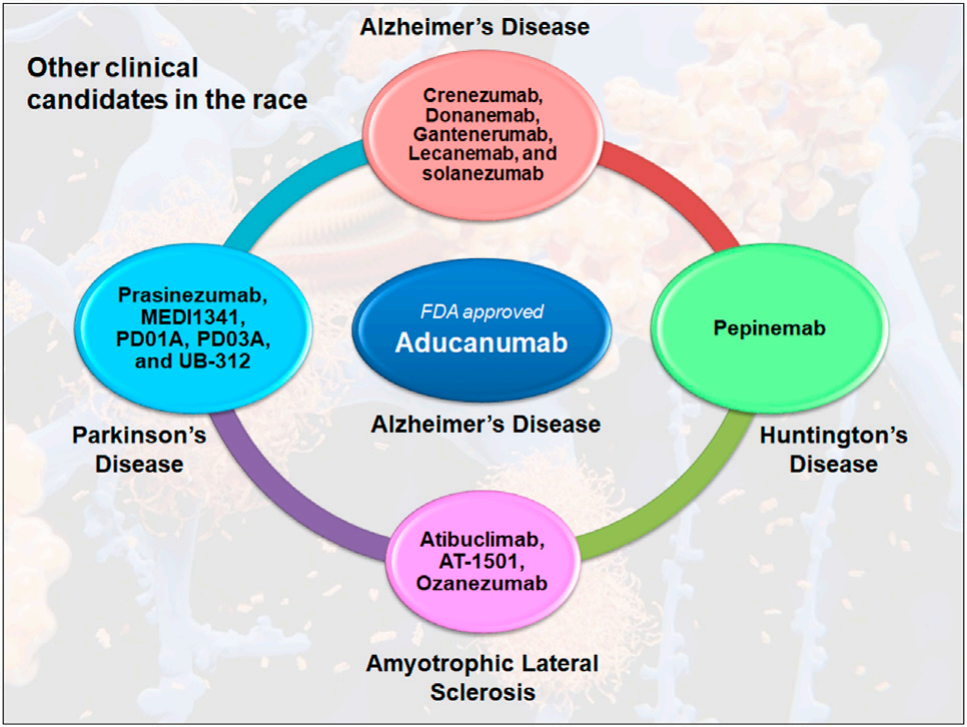
Antibody therapy in the clinical competition for neurodegenerative diseases. Many antibodies are competing for the success in clinical trials, including crenezumab, donanemab, gantenerumab, lecanemab, and solanezumab, which are currently in the third stage of Alzheimer’s disease. In Parkinson’s disease, only prasinezumab is being tested in phase II clinical trials, while other drugs including MEDI1341, PD01A, PD03A and UB312 are in phase I. However, in Huntington’s disease, pepinemab is the only clinical drug candidate that has reached phase II. This is similar to Amyotrophic Lateral Sclerosis, where Atibuclimab (previously known as IC 14), AT1501 and ozezumab are being tested as phase II clinical drug candidates.

The FDA’s recent contentious approval of aducanumab has raised the prospect that the agency would now be more ready to fast-track therapies for a wide range of neurological diseases, including Parkinson’s disease, Huntington’s disease, and Amyotrophic lateral sclerosis **(** Figure 1 **)**. A recent phase II trial of an α-synuclein-targeted antibody failed to make an overall dent in the symptoms of Parkinson’s, Jankovic explains. Still, it delayed the worsening of people’s tremors, stiffness and slowness of movement. Drug-development partners Roche has since launched a larger phase II trial to look at the motor-function benefits of the drug candidate. However, in Huntington’s disease, the most advanced developed drug candidate to lower huntingtin levels is tominersen, developed by Roche and Ionis Pharmaceuticals in Carlsbad, California. But for Urnov, the failure of tominersen is a prime example of why the FDA should not approve drugs using regulatory goalposts such as amyloid plaques or HTT (Mullard, 2021a). Many scientists are concerned that this regulatory precedent may elevate false hope over sound clinical science. They don’t want a future with numerous medications but desire a future with various effective medications that may be prescribed.

## Other Therapeutic Options Should Not Be Overlooked

Ultimately, the approval of an amyloid-targeting medication for AD shows the amyloid hypothesis’s ongoing debate. One risk of pursuing anti-Aβ treatments that haven’t paid off is that other potential concepts may be put on the back burner. Therefore, other therapeutic options must be explored. Anti-tau treatments are also being developed by many firms, as tau is another aggregation-prone protein with an indicative role in the disease’s development. Aside from tau, numerous additional methods are being investigated, including inflammation inhibition. After completing a phase 2b/3 study of the c-kit inhibitor masitinib in mild and moderate AD, AB Science released positive but limited data in December, and another anti-inflammatory therapy, Novo Nordisk’s glucagon-like peptide-1 (GLP1) analog semaglutide, is entering a phase 3 trial early this year. Inspired by studies on youthful blood components that promote brain regeneration, Alkahest and its partner Grifols are also investigating plasma exchange and plasma fractionation techniques in the clinic (Zipkin, 2021).

## Concluding Remarks

Aducanumab is an alluring treatment hoping to help people with early Alzheimer’s disease live longer and healthier lives. It demonstrated the potential of science in solving severe public-health concerns and escorting in a crisis moment in human history. It is anticipated to improve the lives of millions of people with or at risk of neurodegenerative diseases. However, it is important to note that it has nothing to do with targeting the neurodegenerative mechanism of the disease. Still, it may serve as an additive therapy to relieve the burden of neurotoxicity caused by the amyloid species. In addition, any unconventional approach can intercept the process in the really early phases, when neurons are not yet irreversibly compromised are highly regarded as targeted therapies. Therefore, utmost care should be needed for future research endeavors to develop disease-modifying therapies for neurodegenerative diseases. Despite other treatments, theories have shown promise, but none has been investigated as thoroughly as Aβ. In reality, there’s enough data to back it up; however, it’s not the only story.
